# Aligning Antimicrobial Stewardship and Infection Prevention Through Repeated Assessment and Feedback: A Multisite Quality Improvement Initiative in Viet Nam

**DOI:** 10.1093/ofid/ofag605

**Published:** 2026-10-10

**Authors:** Quoc Phuong Nguyen, Van Giang Tran, Hai Yen Nguyen, Thi Phuong Oanh Pham, Trung Cap Nguyen, Minh Dien Vu, Anh Cuong Tran, Thi Ngoc Diep Nguyen, Van Minh Nguyen, Ba Hai Mai, Bao Dung Vo, Thanh Bao Nguyen, Dinh Phu Vu, Thi Thuy Van Pham, Thi Bich Ngoc Hoang, H Rogier van Doorn, Thomas Kesteman, Huong Thi Lan Vu

**Affiliations:** Infectious Diseases, Hanoi Medical University, Hanoi, Viet Nam; Infectious Diseases, National Hospital for Tropical Diseases, Hanoi, Viet Nam; Infectious Diseases, Hanoi Medical University, Hanoi, Viet Nam; Infectious Diseases, National Hospital for Tropical Diseases, Hanoi, Viet Nam; Research Group, Oxford University Clinical Research Unit Hanoi, Hanoi, Viet Nam; Research Group, Oxford University Clinical Research Unit Hanoi, Hanoi, Viet Nam; Infectious Diseases, National Hospital for Tropical Diseases, Hanoi, Viet Nam; Infectious Diseases, National Hospital for Tropical Diseases, Hanoi, Viet Nam; Antimicrobial Stewardship Committee, Viet Nam-Sweden Uong Bi Hospital, Uong Bi, Viet Nam; Antimicrobial Stewardship Committee, Viet Nam-Sweden Uong Bi Hospital, Uong Bi, Viet Nam; Antimicrobial Stewardship Committee, Hue University of Medicine and Pharmacy, Hue University, Hue, Viet Nam; Antimicrobial Stewardship Committee, Hue University of Medicine and Pharmacy, Hue University, Hue, Viet Nam; Antimicrobial Stewardship Committee, Gia Lai Provincial Hospital, Pleiku, Viet Nam; Antimicrobial Stewardship Committee, Gia Lai Provincial Hospital, Pleiku, Viet Nam; Infectious Diseases, National Hospital for Tropical Diseases, Hanoi, Viet Nam; Clinical Pharmacy Department, Hanoi University of Pharmacy, Hanoi, Viet Nam; Microbiology Department, Viet Nam National Children's Hospital, Hanoi, Viet Nam; Research Group, Oxford University Clinical Research Unit Hanoi, Hanoi, Viet Nam; Centre for Tropical Medicine, Nuffield Department of Medicine, University of Oxford, Oxford, UK; Research Group, Oxford University Clinical Research Unit Hanoi, Hanoi, Viet Nam; Centre for Tropical Medicine, Nuffield Department of Medicine, University of Oxford, Oxford, UK; Research Group, Oxford University Clinical Research Unit Hanoi, Hanoi, Viet Nam

**Keywords:** antimicrobial stewardship, hospital, infection prevention and control, quality improvement, Viet Nam

## Abstract

**Background:**

Antimicrobial stewardship (AMS) and infection prevention and control (IPC) are complementary strategies to improve patient safety and address antimicrobial resistance (AMR). In low- and middle-income countries (LMICs), they are often implemented separately, limiting potential impact.

**Objective:**

To evaluate the feasibility of an aligned AMS-IPC improvement approach and describe changes in implementation in Vietnamese hospitals.

**Methods:**

We conducted a quality improvement initiative in 4 hospitals within the national AMR surveillance network in Viet Nam (March–September 2025). We used US-CDC's tools to guide implementation, including the Global Antibiotic Stewardship Evaluation Tool (G-ASET) and the Infection Control Assessment and Response (ICAR) tool. Baseline assessments were followed by feedback, multidisciplinary action planning, and targeted capacity building. Follow-up occurred 2–5 months later. Changes were analyzed descriptively using quantitative scores and qualitative synthesis and reported following the SQUIRE 2.0 guidelines.

**Results:**

All hospitals had established IPC programs at baseline, while AMS maturity varied. G-ASET scores increased across all sites, with larger absolute gains in hospitals starting from lower baselines. Improvements were seen in leadership and governance, education and training, stewardship actions, and monitoring and reporting. Selected IPC activities aligned with AMS priorities also improved, particularly transmission-based precautions, environmental cleaning, and cross-team coordination. Infrastructure-dependent areas, such as water safety, showed limited short-term progress.

**Conclusions:**

This aligned AMS-IPC quality improvement strategy was feasible and associated with improvements in implementation capacity and program maturity over short follow-up. Further studies with longer follow-up, comparator designs, and outcome-level measures are needed to evaluate effectiveness, sustainability, and generalizability.

## Teaser

Repeated assessment and feedback can help bring antimicrobial stewardship and infection prevention into closer alignment. This multisite quality improvement initiative in Viet Nam shows the potential of integrated, data-driven approaches to improve hospital practice.

## Graphical Abstract

**Graphical Abstract:**
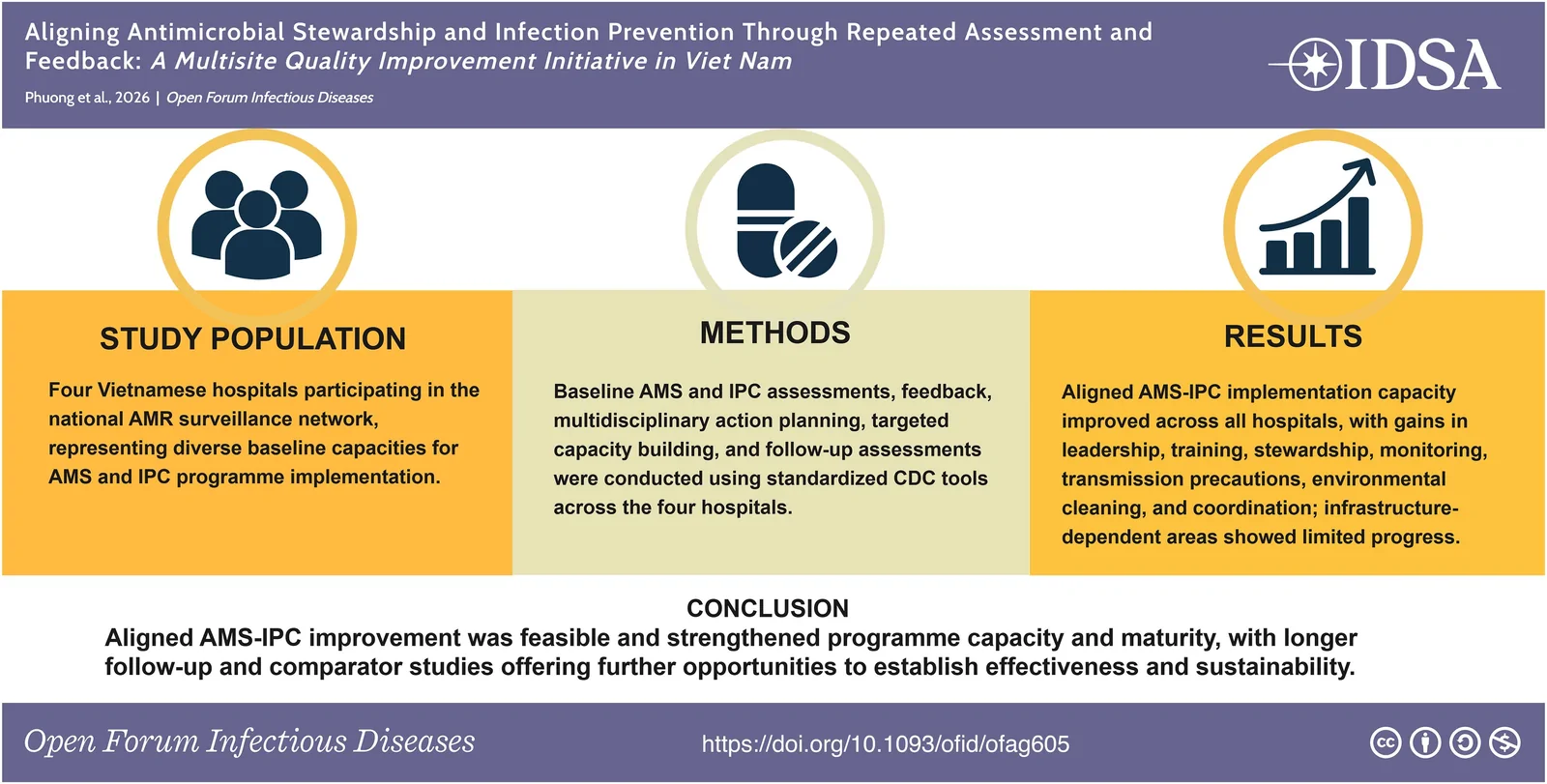
This graphical abstract is also available at Tidbit: https://tidbitapp.io/tidbits/aligning-antimicrobial-stewardship-and-infection-prevention-through-repeated-assessment-and-feedback-a-multisite-quality-improvement-initiative-in-viet-nam?utm_campaign=tidbitlinkshare&utm_source=IO

Key messages

- Repeated assessment and structured feedback within an AMS-IPC quality improvement approach were associated with improved AMS implementation across diverse Vietnamese hospitals.
- Use of G-ASET and ICAR helped hospitals identify common system gaps and develop coordinated, locally tailored improvement plans.
- Gains were most evident in domains influenced by leadership, coordination, and behavior change, whereas areas requiring financial investment showed less short-term improvement.
- This assessment-guided network approach may inform future scale-up of activities to support AMS-IPC alignment in LMIC hospitals and guide future Fleming Fund-type investments.

## INTRODUCTION

### Problem Description

Antimicrobial resistance (AMR) poses a major threat to patient safety and healthcare quality worldwide, with hospitals in low- and middle-income countries (LMICs) disproportionately affected. In hospital settings, inappropriate antimicrobial use and healthcare-associated infections (HAIs) interact to accelerate the emergence and spread of resistant organisms. Antimicrobial stewardship (AMS) and infection prevention and control (IPC) are core quality and safety strategies for addressing AMR and its consequences [1–3].

Despite this interdependence, AMS and IPC are frequently implemented as separate programs, resulting in fragmented efforts, duplication of activities, and missed opportunities for system-level improvement [4]. This disconnect is particularly evident in LMICs, where IPC programs are more widely established than AMS but are often under-resourced, inconsistently implemented, and limited by hospital infrastructure. Alignment is further constrained by weak organizational structures, limited surveillance capacity, and workforce and diagnostic gaps [5]. Consequently, both IPC and AMS remain incompletely implemented, with few integrated models in practice and limited evidence on their combined impact, underscoring the need for stronger, system-level coordination [6, 7].

In Viet Nam, national action plans on AMR and Ministry of Health guidelines mandate the establishment of multidisciplinary AMS committees and IPC programs in hospitals [8–12]. However, implementation remains uneven. Studies from Viet Nam and other Southeast Asian countries have identified common barriers, including limited dedicated resources, insufficient training, weak governance structures, and suboptimal collaboration between clinical teams, pharmacy, microbiology, and IPC units [13–16]. As a result, AMS activities are often confined to guideline development or reporting requirements, while IPC efforts focus primarily on compliance with infection control standards, with limited coordination between the two.

Evidence further shows that although healthcare workers generally possess adequate IPC knowledge, adherence to recommended practices is variable, reflecting a well-described “know-do gap” [16, 17]. Structural constraints, including infrastructure and design, overcrowding, limited microbiology capacity, and workforce shortages, further hinder effective implementation [17, 18]. Assessments of IPC capacity across Vietnamese hospitals reveal heterogeneity in systems, lack of standardized procedures, and weak coordination between clinical and IPC teams [19]. These gaps contribute to ongoing transmission of HAIs and high levels of AMR, particularly in high-risk settings such as ICUs [20].

### Rationale

International guidance increasingly emphasizes the alignment of AMS and IPC as part of a systems-based quality improvement approach [3, 21]. Such alignment has the potential to address shared drivers of AMR, including leadership engagement, workforce capacity, surveillance, and management of priority AMR pathogens, more effectively than standalone interventions. However, evidence on how to operationalize AMS-IPC alignment in routine hospital practice in LMICs is limited, particularly across hospitals with varying baseline capacity and resource constraints [15, 22, 23].

Viet Nam has developed a national AMR surveillance network, strengthened through international support, including from the Fleming Fund (2015–2025), which has enhanced laboratory capacity and expanded the availability of surveillance data [24, 25]. Building on this foundation, there is a need to shift focus from data generation to implementation, supporting hospitals to translate surveillance and assessment findings into coordinated, locally relevant actions. Quality improvement approaches that combine repeated assessment, structured feedback, and context-adapted action planning offer a pragmatic pathway to support this alignment, yet their feasibility and impact in real-world LMIC hospital settings remain underexplored.

### Aim

The aim of this multisite quality improvement initiative was to assess the feasibility of implementing an aligned AMS-IPC quality improvement approach in routine hospital settings in Viet Nam and to evaluate changes in AMS and IPC implementation following structured assessment, feedback and targeted capacity-building activities.

While the Fleming Fund has strengthened AMR surveillance capacity in Viet Nam, its conclusion without a subsequent phase [26, 27] underscores the importance of sustainable, facility-level approaches to translate surveillance and assessment capacity into routine implementation and local action.

## METHODS

### Study Design

This study reports a multisite quality improvement initiative using repeated assessment, structured feedback, and capacity-building interventions to support alignment between AMS and IPC. The project was implemented within the Fleming Fund Phase II Country Grant in Viet Nam and followed principles of the SQUIRE 2.0 reporting guidelines for quality improvement studies [28] (Supplementary Data, Table 1).

The intervention used 2 complementary assessment tools:

- Global Antibiotic Stewardship Evaluation Tool (G-ASET): a structured, facility-level assessment instrument developed by the US CDC with the involvement of the G-ASET Development and Implementation Group in Asia [29–32] that evaluates the implementation of AMS programs across core domains, using standardized indicators aligned with international AMS frameworks to identify gaps and guide targeted improvement.
- Infection Control Assessment and Response (ICAR): a practice-oriented and operational assessment framework developed by the US CDC to evaluate IPC capacity and practices across healthcare settings. Through structured review of policies, practices, and infrastructure using standardized closed- and open-ended questions, ICAR identifies implementation strengths and gaps across key IPC domains in alignment with WHO core components, providing a basis for feedback and quality improvement planning [33, 34].

Both tools were applied at baseline and follow-up to guide local action planning and assess changes over time.

### Setting and Participating Hospitals

The project was conducted between March and September 2025 in 4 hospitals within the Viet Nam Antimicrobial Resistance Surveillance Network, supported by the Fleming Fund. The hospitals were purposively selected to represent diverse healthcare contexts and baseline AMS and IPC implementation capacity:

- National Hospital for Tropical Diseases (NHTD): a tertiary referral hospital specializing in infectious diseases.
- Viet Nam-Sweden Uong Bi Hospital (VSUH): a provincial general hospital in Quang Ninh province.
- Gia Lai Provincial General Hospital (GLPH): a provincial general hospital in the Central Highlands.
- Hue University of Medicine and Pharmacy Hospital (UMPH): an academic general hospital in Central Viet Nam.

As the sites were purposively selected from the Viet Nam Antimicrobial Resistance Surveillance Network to represent diverse healthcare contexts and baseline AMS and IPC capacity, the findings may not be generalizable to hospitals without prior AMR program engagement or external technical support. All 4 hospitals had existing IPC structures and microbiology services, but AMS programs were at varying stages of development at baseline. Two sites have previously been engaged in our AMS assessment research (2022–2023) at Oxford University Clinical Research Unit (OUCRU)—Viet Nam.

### Intervention Overview: Aligned AMS-IPC Quality Improvement Approach

For this study, AMS-IPC alignment was operationalized as shared governance, coordinated planning, and joint review of assessment findings. The study evaluated whether this quality improvement strategy improved implementation capacity and program alignment; sustained integration was not assessed as a separate outcome. The intervention was designed as an aligned AMS-IPC quality improvement cycle with 4 core components: (1) baseline assessment of AMS and IPC capacity using G-ASET and ICAR, (2) structured feedback to hospital leadership and multidisciplinary teams, (3) locally adapted action planning and capacity-building activities, and (4) follow-up assessment to document implementation progress and changes in program maturity. The approach was intended to promote greater alignment between AMS and IPC activities through shared governance, coordinated action planning, and joint review processes, particularly in relation to priority AMR pathogens (defined by WHO) [35], surveillance, and infection prevention practices. Overall timeline and activities are illustrated in Figure 1 and Supplementary Table 2; Supplementary Table 3 summarizes the site-specific quality improvement components and support intensity to aid reproducibility.

**Figure 1.**
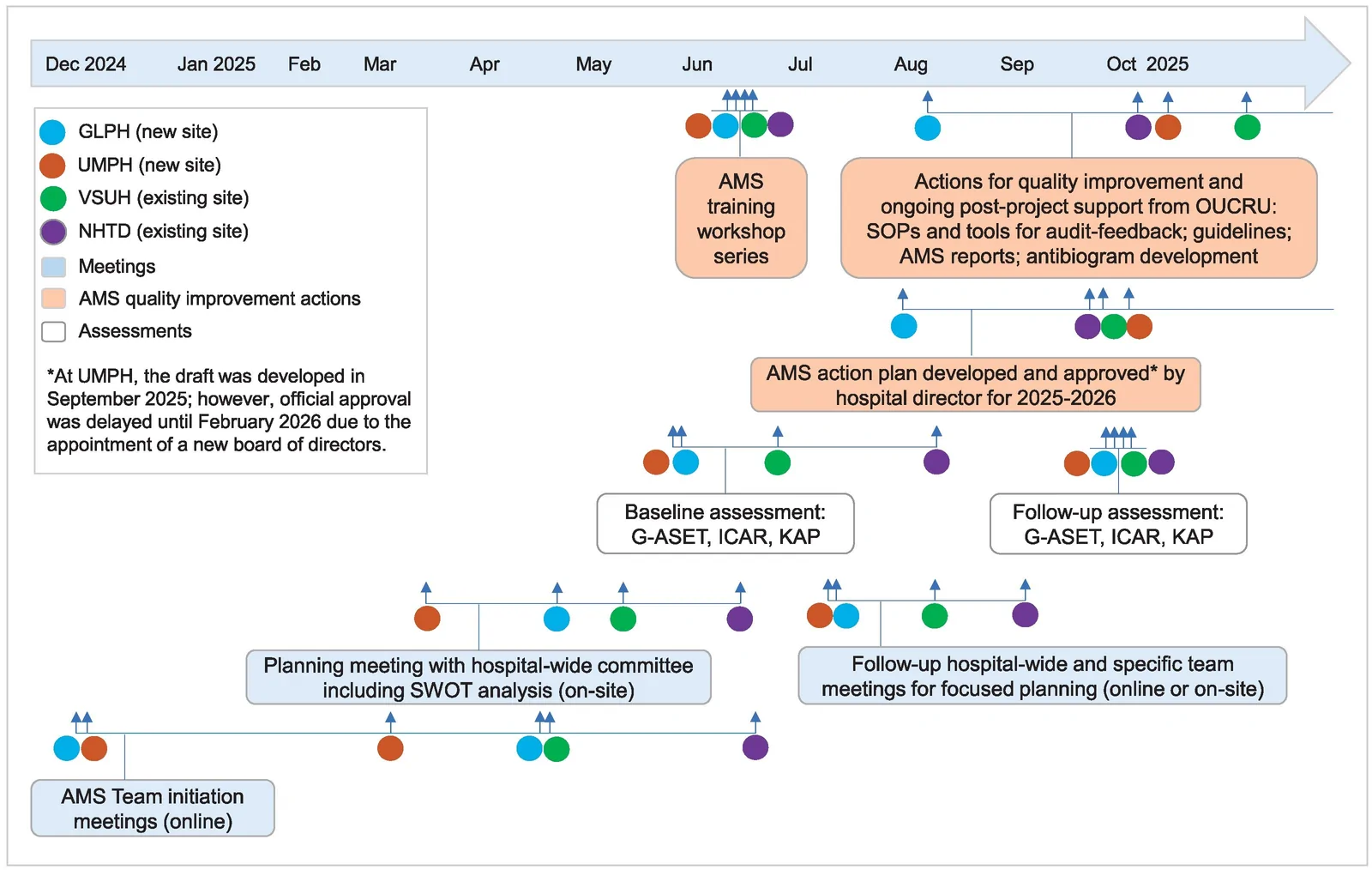
Timeline of AMS-IPC activities across 4 hospitals, December 2024–October 2025. The figure shows AMS team initiation meetings, hospital-wide planning meetings with SWOT (strengths, weaknesses, opportunities, and threats) analysis, baseline assessments using G-ASET, ICAR, and KAP (knowledge, attitudes, and practices) tools, AMS training workshops, development and approval of site-specific AMS action plans, post-project quality-improvement support from the Oxford University Clinical Research Unit (OUCRU), and follow-up assessments using the same tools.

### AMS Assessment: Global Antibiotic Stewardship Evaluation Tool

AMS was assessed using the G-ASET in 5 core domains of hospital AMS programs:

- Leadership commitment and accountability
- Resources
- Education and training
- Specific AMS actions
- Tracking, monitoring, and reporting

The tool consists of 66 structured questions, with responses scored as not implemented (0), partially implemented (2.5), or fully implemented (5).

Domain scores and overall scores were calculated for each hospital at baseline and follow-up. The G-ASET assessment was conducted through facilitated self-assessment meetings involving representatives from hospital leadership, clinical departments, pharmacy, microbiology, IPC, and quality management. To contextualize quantitative scores, semi-structured discussions with the research team were held during and after the assessment to explore barriers, facilitators, and contextual factors influencing AMS implementation. In addition, a knowledge-attitudes-practices (KAP) survey was administered by each hospital using the form developed under US CDC's initiative for the G-ASET development [32] (Supplementary Figure 1). The KAP survey was used formatively to guide training priorities and support discussion during AMS team meetings rather than as a prespecified outcome measure.

### IPC Assessment: Infection Control Assessment and Response

IPC capacity and practices were assessed using the ICAR tool, adapted with a focus on the following domains under this quality improvement initiative:

- IPC program structure and governance
- Hand hygiene
- Transmission-based precautions
- Environmental cleaning and disinfection
- Water safety and environmental sources of infection

ICAR assessments were conducted onsite by the hospital IPC teams, with support from the study research assistant. The assessment combined document review, direct observation, and structured interviews with IPC personnel and clinical staff.

Following each assessment, hospitals received written and verbal feedback summarizing strengths, identified gaps, and priority improvement actions. Feedback was aligned with national Ministry of Health regulations and international US CDC and WHO guidance. As the ICAR tool was adapted to focus on the IPC components most relevant to the participating sites and the AMS-IPC alignment initiative, findings are presented descriptively and are not intended for formal cross-site comparison.

### Action Planning and Capacity-Building Activities

Following baseline assessments, each hospital participated in multidisciplinary action-planning meetings involving hospital leadership, AMS committees, IPC teams, pharmacy, microbiology, and clinical departments.

Action plans were developed in alignment with

- Viet Nam Ministry of Health (2020) Decision 5631/QĐ-BYT on hospital AMS implementation,
- WHO practical toolkit for AMS in LMICs (WHO 2019), and
- National IPC regulations (2017, 2018).

Capacity-building activities during the intervention period included (more detail in Supplementary Method S1)

- targeted AMS training workshops (clinical pharmacy, microbiology, management of common infections),
- provision of AMS standard operating procedures and information materials,
- support for development or revision of hospital-level AMS-IPC action plans, and
- tools to support prospective audit and feedback, including data collection templates.

AMS and IPC priorities were aligned, particularly for management of priority AMR pathogens, surveillance and reporting, isolation and transmission-based precautions, environmental and water safety measures influencing infection risk and antibiotic use.

### Follow-up Assessment

Follow-up G-ASET and ICAR assessments were conducted approximately 2–5 months after baseline, depending on hospital scheduling and availability of key personnel (Figure 1). The follow-up assessments aimed to document changes in selected AMS and IPC implementation practices, assess progress against locally defined action plans, and identify persistent barriers and contextual constraints. Changes were evaluated descriptively by comparing baseline and follow-up findings within each hospital.

### Data Analysis

Quantitative data from G-ASET were summarized using descriptive statistics, including domain-specific and overall scores at baseline and follow-up. Because G-ASET scores are facility-level program implementation measures rather than sampled observations, confidence intervals were not calculated; results are presented descriptively as baseline and follow-up scores and absolute percentage-point changes. Qualitative information from ICAR feedback, action-planning discussions, and assessment debriefs were synthesized descriptively to contextualize implementation findings, focusing on governance and leadership, workforce and training, alignment between AMS and IPC activities, and contextual facilitators and barriers. Given the quality improvement design, no hypothesis testing or causal inference was undertaken.

### Ethical Considerations

This project was conducted as a quality improvement initiative to support implementation of national AMS and IPC programs. Ethical approval was obtained from the Ethics Review Committee at the University of Oxford (OxTREC- 602-24) and NHTD Institutional Review Board (Decision 01-2025/HĐĐĐ-NĐTƯ). Participation was endorsed by hospital leadership, and no patient-level identifiable data were collected.

## RESULTS

### Baseline Characteristics of Participating Hospitals

The 4 participating hospitals represented diverse healthcare contexts and baseline levels of AMS and IPC implementation (Table 1). National Hospital for Tropical Diseases, a tertiary infectious disease referral center, had the most established AMS and IPC structures, including strong leadership engagement, multidisciplinary committees, and routine use of microbiology data to inform clinical and infection control decisions. The remaining 3 hospitals were provincial or academic general hospitals with existing AMS and IPC committees but more variable implementation capacity. At baseline, these hospitals had foundational governance structures in place but limited dedicated AMS resources, inconsistent formalization of AMS activities, and uneven alignment between AMS and IPC practices, particularly in education, surveillance, and coordinated management of priority AMR pathogens (Table 2). Additional baseline antibiotic-use context for each hospital is provided in Supplementary Table 4.

**Table 1.** Baseline Characteristics of Participating Hospitals

Hospital	Level/Type	Geographic Location	Primary Clinical Focus	AMS Program Status at Baseline	IPC Program Status at Baseline	Prior AMS/IPC Assessment Experience
NHTD	Tertiary referral hospital	North Viet Nam	Infectious diseases	Established AMS program with multidisciplinary committee and prior action plans	Established IPC program with dedicated department and surveillance	Prior AMS assessments (US CDC initiative); no formal ICAR before this project
VSUH	Provincial general hospital	North Viet Nam	General acute care	Formal AMS program in place but variable implementation in recent years	Established IPC program with hospital-wide network	Prior AMS assessment (2023); no formal ICAR before this project
GLPH	Provincial general hospital	Central Highlands	General acute care	No formal AMS program at baseline; antibiotic-related activities conducted without structured governance	Established IPC program with routine practices	No prior formal AMS or ICAR assessment
UMPH	Academic general hospital	Central Viet Nam	General and teaching hospital	No formal AMS program at baseline; partial stewardship activities embedded in clinical practice	Established IPC program with variable operational maturity	No prior formal AMS or ICAR assessment

All hospitals had established AMS and IPC structures at baseline, although the level of operational maturity and prior exposure to formal AMS or IPC assessments varied across sites.

**Table 2.** Baseline Level of AMS and IPC Programs Assessed Using the Global Antibiotic Stewardship Evaluation Tool (G-ASET) and the Infection Control Assessment and Response (ICAR) Tool. Baseline Levels Reflect Overall Patterns Across Hospitals Rather Than Individual Site Scores

Domain	Assessment Tool	Baseline Level (Overall)	Evidence From Baseline Assessments
Leadership and governance (AMS)	G-ASET	Moderate to high	AMS committees existed at all sites, but formal action plans and regular meetings were absent or inconsistent in 3 hospitals
Resources and workforce (AMS)	G-ASET	Moderate	Limited dedicated AMS staff and no protected budgets at most hospitals
Education and training (AMS)	G-ASET	Low to moderate	Lowest-scoring AMS domain at baseline, particularly in provincial hospitals
Specific AMS actions	G-ASET	Moderate to high	Treatment guidelines and formulary controls present, but prospective audit with feedback to prescribers remained limited; antibiograms were incomplete and not fully aligned with CLSI guidelines in all hospitals
Tracking, monitoring, and reporting (AMS)	G-ASET	Moderate	Routine reporting existed, but feedback to prescribers and action planning were limited
IPC program structure	ICAR	Moderate to high	IPC departments and committees were established at all hospitals
Hand hygiene	ICAR	High	Infrastructure and supplies were generally adequate, but supply assurance, nail policies, hand-care support, and adherence monitoring varied across sites
Transmission-based precautions	ICAR	Moderate	Gaps existed in signage standardization, role clarity, and response to priority AMR pathogens
Environmental cleaning	ICAR	Moderate	Cleaning governance varied; 2 hospitals lacked formal product selection processes, and 2 lacked minimum cleaning-time procedures
Water safety	ICAR	Low	Least mature IPC domain, with limited formal water safety programs

Low, moderate, and high are qualitative descriptors summarizing overall baseline patterns across hospitals and were not assigned using predefined numerical thresholds; raw G-ASET scores are reported separately in Table 3.

### Baseline AMS Implementation Assessed by G-ASET

At baseline, overall G-ASET scores demonstrated substantial variation in AMS program maturity across the 4 hospitals (Table 3). NHTD had the highest overall baseline score (84.9%), reflecting a well-established AMS program with strong leadership engagement and routine stewardship activities. VSUH showed a moderate baseline score (78.0%), indicating the presence of a formal AMS structure but inconsistent implementation in recent years. GLPH and UMPH had lower baseline scores (both 73.5%), consistent with the absence of formally structured AMS programs despite the presence of selected antibiotic-related activities. Across all hospitals, baseline scores were lowest in the domains of education and training and resources, highlighting common system-level gaps that informed subsequent capacity-building priorities.

**Table 3.** Overall AMS Program Implementation Scores at Baseline and Follow-up Across 4 Hospitals, Assessed Using G-ASET

Hospital	Baseline G-ASET Overall Score (%)	Follow-up G-ASET Overall Score (%)	Absolute Change (Percentage Points)	Time Between Assessments
NHTD	84.9	86.4	+1.5	2 m
VSUH	78.0	95.5	+17.5	3 m
GLPH	73.5	90.1	+16.6	5 m
UMPH	73.5	88.6	+15.1	5 m

All hospitals demonstrated increases in overall scores, with larger improvements observed at sites with lower baseline maturity.

Domain-level analysis of baseline G-ASET assessments revealed a consistent pattern of strengths and gaps across the 4 hospitals. Leadership commitment and accountability scored relatively high, reflecting the presence of AMS committees and senior management recognition of stewardship as a priority, although regular committee meetings and formally endorsed action plans were absent or inconsistently implemented in 3 hospitals. Specific AMS actions, including the existence of treatment guidelines, formularies, and selected stewardship activities, were also comparatively well developed at baseline. In contrast, education and training represented the weakest domain across sites, particularly in provincial hospitals, with limited structured AMS training for clinical staff, students, and trainees. Resource-related gaps were also evident, including a lack of dedicated AMS personnel, protected time, and formal budget allocation. Tracking, monitoring, and reporting activities were present in all hospitals but were often focused on routine reporting requirements, with limited use of audit findings, feedback to prescribers, or systematic action planning to optimize antibiotic use.

### Baseline IPC Implementation Assessed by ICAR

Baseline ICAR assessments indicated that all 4 hospitals had established IPC programs, including formal committees or networks, designated staff, written IPC procedures, access to electronic medical records, and routine IPC risk-assessment activities. Hand hygiene was the most consistently established domain: all hospitals reported alcohol-based hand rub use in most clinical situations and soap-and-water handwashing for visibly soiled hands. Standard healthcare-worker hand hygiene indications were reported across sites, although supply assurance mechanisms, nail-related policies, staff hand-care support, and monitoring of adherence varied. Transmission-based precautions, particularly for priority AMR pathogens, were reported across hospitals but varied in implementation, including differences in signage, role clarity, and isolation capacity. One hospital reported reliance on cohorting and care sequencing when single rooms were unavailable. Environmental cleaning systems were present, but governance was uneven; 2 hospitals lacked formal cleaning-product selection processes, and 2 lacked minimum cleaning-time procedures. Water safety was the least mature IPC domain: only 1 hospital had a clearly dated water-related IPC risk assessment, 3 lacked sink-drain biofilm-reduction measures, and none had procedures for planned water-supply interruption. These baseline IPC gaps, particularly in transmission control and environmental domains, were identified as priorities for alignment with AMS interventions during subsequent integrated action planning.

Taken together, baseline G-ASET and ICAR assessments showed that while foundational governance structures for both AMS and IPC were present across all hospitals, important operational gaps persisted, particularly in education, workforce capacity, feedback mechanisms, and standardization of transmission-based and environmental controls. These convergent weaknesses highlighted the interdependence of AMS and IPC and provided a clear rationale for an approach that aligned stewardship activities with infection prevention priorities.

### Aligned AMS-IPC Action Planning and Implementation

Following baseline assessments, all 4 hospitals undertook multidisciplinary action-planning processes that brought AMS and IPC priorities together. Action-planning meetings involved hospital leadership, AMS committees, IPC teams, pharmacy, microbiology, and clinical departments and were guided by findings from both G-ASET and ICAR assessments. These discussions enabled hospitals to prioritize system-level gaps that affected both antibiotic use and infection transmission, particularly governance, workforce capacity, education, surveillance, and management of priority AMR pathogens.

All hospitals developed context-specific AMS action plans that incorporated IPC-related activities, including strengthened collaboration between AMS and IPC teams, assignment of IPC representatives to AMS committees, and alignment of stewardship activities with transmission-based precautions for priority AMR pathogens and environmental hygiene practices. Three hospitals (NHTD, VSUH, and GLPH) obtained formal approval of their AMS action plans from hospital leadership during the project period, while the action plan at the remaining hospital was finalized but its approval was delayed by 5 months because of ongoing administrative transitions. Across sites, action plans commonly prioritized establishment or strengthening of core AMS teams, regular committee meetings, structured education and training, review and updating of treatment guidelines, prospective audit and feedback, improved use of microbiology data and antibiograms, and enhanced coordination with IPC activities such as hand hygiene monitoring, cohorting and isolation practices, and surveillance of hospital-acquired infections.

Capacity-building activities supported implementation of these integrated plans and included targeted AMS training workshops, dissemination of standard operating procedures, and provision of tools to support prospective audit and feedback and routine monitoring. Alignment between AMS and IPC was supported through shared governance mechanisms, joint review of surveillance data, and coordinated feedback to clinical teams, particularly for infections caused by priority AMR pathogens. These processes formed part of a structured quality improvement approach, laying the foundation for improvements observed at follow-up and enabling hospitals to tailor AMS and IPC activities to their local contexts. The intervention was delivered through a multi-hospital network, enabling shared learning, peer comparison, and coordinated capacity building across sites.

### Changes in AMS and IPC Implementation at Follow-up

Follow-up assessments conducted 2–5 months after baseline showed improvements in AMS implementation capacity and program maturity across all 4 hospitals, with the magnitude of change varying by baseline status and local context. G-ASET scores increased at all 4 hospitals between baseline and follow-up, with the larger absolute gains observed at sites with lower baseline maturity (Table 3).

Domain-specific analysis (Table 4) suggested improvements in leadership and governance, education and training, and tracking and reporting. Hospitals reported more regular AMS committee meetings, clearer role assignment, and formalization of annual action plans. Education and training scores increased following targeted AMS workshops, particularly in hospitals with limited structured training at baseline. Improvements were also documented in tracking, monitoring, and reporting, including greater use of prescription audits, development or updating of antibiograms, and increased feedback of stewardship findings to clinical teams. Persistent challenges remained in securing dedicated financial resources and protected staff time for AMS activities, particularly in provincial hospitals.

**Table 4.** Baseline and Follow-up Changes in AMS and IPC Domains, Assessed Using G-ASET and ICAR

Domain	Assessment Tool	Baseline Status	Follow-up Status	Direction of Change
Leadership and governance (AMS)	G-ASET	Moderate to high; committees present, but action plans inconsistently formalized	High; clearer roles, regular meetings, and formal action plans approved or finalized	↑
Resources and workforce (AMS)	G-ASET	Moderate; limited dedicated staff and no protected budgets	Moderate; improved access to tools and systems but persistent budget and staffing constraints	↑ (limited)
Education and training (AMS)	G-ASET	Low to moderate; limited structured AMS training	Moderate to high; targeted workshops and improved staff engagement	↑
Specific AMS actions	G-ASET	Moderate to high; guidelines and formulary controls present	High; strengthened audit, feedback, and use of microbiology data	↑
Tracking, monitoring, and reporting (AMS)	G-ASET	Moderate; routine reporting with limited feedback	High; improved audits, use of antibiograms, and reporting to clinicians	↑
IPC program structure	ICAR	Moderate to high; IPC committees established	High; clearer coordination with AMS and clinical teams	↑
Hand hygiene	ICAR	High; infrastructure largely adequate	High; incremental improvements in monitoring and feedback	↑ (limited)
Transmission-based precautions	ICAR	Moderate; inconsistent signage and practices for priority AMR pathogens	Moderate to high; improved standardization and coordination	↑
Environmental cleaning	ICAR	Moderate; variable governance and auditing	Moderate to high; improved oversight and feedback	↑
Water safety	ICAR	Low; limited formal programs	Low to moderate; minimal short-term change	→

ICAR was adapted to focus on the IPC components most relevant across participating sites and to AMS-IPC alignment in this quality improvement initiative; therefore, findings are presented descriptively, and no standardized ICAR scores were calculated.

Hospitals also reported that improvements were observed in several IPC practices at follow-up, particularly in areas aligned with AMS priorities. These included more consistently documented and reported implementation of transmission-based precautions, enhanced coordination between IPC, microbiology, and clinical teams for management of priority AMR pathogens, and more structured feedback on hand hygiene and environmental practices. While foundational IPC structures remained stable, incremental gains were noted in standardization of isolation practices and environmental cleaning oversight. Water safety practices showed limited short-term change, reflecting structural and infrastructural constraints beyond the project timeframe.

Overall, follow-up findings suggest improvements in selected AMS-IPC domains following the implementation of aligned action planning and capacity-building activities, particularly in areas requiring governance, coordination, and behavioral change rather than major infrastructure investment.

## DISCUSSION

This multisite quality improvement initiative suggests that an approach aligning AMS and IPC activities [3, 21], supported by repeated assessment, structured feedback, and targeted capacity building, was associated with improvements in hospital AMS implementation capacity and program maturity across diverse healthcare settings in Viet Nam. By applying complementary assessment tools (G-ASET and ICAR), hospitals were able to identify convergent gaps in governance, workforce capacity, education, and feedback mechanisms and translate these into locally adapted action plans that aligned stewardship activities with infection prevention priorities. Improvements were greatest in hospitals with lower baseline status of AMS implementation and more modest in an already well-established tertiary center, underscoring the importance of contextualizing quality improvement efforts according to baseline capacity and system readiness. The smaller absolute gain at NHTD likely reflects a ceiling effect, as this hospital had the highest baseline AMS maturity and therefore less measurable room for short-term improvement on a facility-level implementation instrument.

A key contribution of this work is showing how alignment between AMS and IPC activities can be supported in LMIC hospital settings, consistent with previous literature on the value of coordinated governance and implementation. Rather than maintaining separate and siloed programs, the intervention promoted shared governance, coordinated action planning, and alignment of surveillance, training, and clinical feedback processes, particularly for the management of priority AMR pathogens [13, 36]. Gains were most pronounced in domains driven by leadership engagement, coordination, and behavioral change, while areas requiring substantial financial investment, such as water safety and diagnostic capacity, showed limited short-term progress [7, 15]. This highlights the need for differentiated expectations and timelines when evaluating AMS-IPC quality improvement interventions.

Compared with standalone AMS or IPC approaches, the aligned AMS-IPC quality improvement strategy may help hospitals address cross-cutting constraints affecting both programs, including weak governance, insufficient training, and limited feedback to clinicians. These challenges have been widely reported across LMIC settings, where gaps in infrastructure, diagnostics, and workforce capacity hinder implementation [5, 15, 37]. Positioning stewardship within broader patient safety and infection control systems may increase the relevance and uptake of AMS activities, while IPC practices aligned with AMS priorities benefited from stewardship input and leadership engagement, reinforcing the bidirectional relationship between antibiotic use and infection prevention [6].

The Fleming Fund investment played an enabling role by providing technical tools and training, as well as a structured framework for reflection, prioritization, and accountability. By leveraging existing surveillance and laboratory capacity, the project focused on strengthening implementation processes at the facility level. Consistent with prior evidence from Viet Nam and the wider Southeast Asian region, governance, leadership, and education emerged as key enablers of AMS implementation [14, 25], while resource constraints, limited diagnostics, and workforce challenges remained persistent barriers [5, 15]. Similar investments across Fleming Fund-supported countries have prioritized strengthening AMR surveillance, laboratory capacity, and data systems, while evidence of facility-level AMS-IPC alignment using structured quality improvement approaches remains limited [38, 39]. The network-based approach [24] supported cross-site learning and peer comparison, which may have facilitated uptake of selected practices more rapidly than is often reported in single-site or short-term interventions.

Strengths of this study include its multisite design, use of established assessment tools, and intentional alignment of AMS and IPC activities within a quality improvement framework. Limitations include the absence of a control group, short and variable follow-up periods, reliance on partly self-assessed data, and the inability to attribute observed improvements to downstream clinical or antimicrobial resistance outcomes within the project timeframe. Because G-ASET is an item-based assessment of the presence or absence of specific AMS components and actions, repeated assessment is unlikely to inflate scores through a learning effect; if anything, follow-up scoring may become more stringent as teams better understand program maturity. Nevertheless, the facilitated self-assessment format and the involvement of hospital teams in ICAR may have introduced social desirability bias, observer bias, and response shift. The absence of independent external assessors is therefore a limitation.

In addition, the intervention was designed to promote greater alignment between AMS and IPC activities through shared governance structures, coordinated planning, and joint review processes. The degree to which this resulted in sustained programmatic integration warrants further prospective evaluation. This study did not measure antimicrobial consumption, prescribing quality, healthcare-associated infections, multidrug-resistant organism transmission, hand hygiene compliance, device-associated infection rates, patient outcomes, or costs. Accordingly, the findings should be interpreted as evidence of improved implementation capacity, and observed changes should be interpreted as reported improvements in implementation capacity rather than externally verified program performance or downstream clinical or public health effectiveness. Finally, the purposive selection of hospitals from an established AMR surveillance network may limit generalizability to district hospitals or other facilities with less baseline AMS/IPC capacity and fewer implementation resources.

Overall, these findings suggest that aligned AMS-IPC strengthening, guided by repeated assessment and feedback, is feasible in hospitals with foundational structures in place. This report is framed as a contextualized quality improvement initiative rather than a comparative study between hospitals; therefore, follow-up findings are interpreted within each site's local context and baseline capacity. For national programs and Fleming Fund-type investments, relatively modest, targeted support for implementation processes, alongside existing investments in surveillance and laboratory capacity, may support improvements in AMS-IPC alignment and contribute to more sustainable responses to antimicrobial resistance in LMICs. Our findings align with WHO guidance emphasizing integration of AMS and IPC activities in resource-limited settings and extend previous work from Viet Nam and Southeast Asia demonstrating the feasibility of stewardship programs despite persistent resource constraints [13, 14, 21]. Sustainability of this approach beyond external facilitation remains uncertain. Continued implementation will likely depend on embedding AMS and IPC activities into routine hospital governance, maintaining protected staff time, sustaining audit-and-feedback and reporting processes, and securing local and national financing mechanisms to support ongoing coordination. Future sustainability may be strengthened by integrating these activities into hospital quality systems and national AMR program support rather than relying on project-based facilitation.

In conclusion, this multisite quality improvement initiative suggests that aligning AMS and IPC through repeated assessment, structured feedback, and context-adapted action planning is feasible and associated with improvements in hospital AMS implementation capacity and program maturity across diverse settings in Viet Nam. By leveraging complementary assessment tools and a network-based support model, hospitals were able to translate surveillance capacity and policy guidance into coordinated, locally relevant actions. The findings suggest that investments targeting governance, workforce capacity, and implementation processes may be particularly valuable, alongside infrastructure investments. This approach may inform future scale-up of activities for strengthening hospital responses to antimicrobial resistance in LMICs and offers important lessons for national programs and Fleming Fund-type investments seeking sustainable impact.

## Supplementary Material

ofag605_Supplementary_Data

## Supplementary Data

Supplementary materials are available at *Open Forum Infectious Diseases* online. Consisting of data provided by the authors to benefit the reader, the posted materials are not copyedited and are the sole responsibility of the authors, so questions or comments should be addressed to the corresponding author.
