# Supplementary material for: Aligning Antimicrobial Stewardship and Infection Prevention Through Repeated Assessment and Feedback: A Multisite Quality Improvement Initiative in Viet Nam
Source: Open Forum Infect Dis. 2026 Oct 10;13(10):ofag605. doi: 10.1093/ofid/ofag605 (PMC13653215; doi:10.1093/ofid/ofag605)
Supplement: Supplementary file 1 [file ofag605_supplementary_data.doc]

**Supplementary Data**

Manuscript: **Aligning Antimicrobial Stewardship and Infection Prevention Through Repeated Assessment and Feedback: A Multisite Quality Improvement Initiative in Viet Nam**

Authors: Nguyen Quoc Phuong^1,2^, Tran Van Giang^1,2^, Nguyen Hai Yen^3^, Pham Thi Phuong Oanh^3^, Nguyen Trung Cap^2^, Vu Minh Dien^2^, Tran Anh Cuong^4^, Nguyen Thi Ngoc Diep^4^, Nguyen Van Minh^5^, Mai Ba Hai^5^, Vo Bao Dung^6^, Nguyen Thanh Bao^6^, Vu Dinh Phu^2^, Pham Thi Thuy Van^7^, Hoang Thi Bich Ngoc^8^, H Rogier van Doorn^3,9^, Thomas Kesteman^3,9^, Vu Thi Lan Huong^3*^

^*^Corresponding author

Author affiliations

1. Hanoi Medical University, Viet Nam
2. National Hospital for Tropical Diseases, Viet Nam
3. Oxford University Clinical Research Unit Hanoi, Viet Nam
4. Viet Nam-Sweden Uong Bi Hospital, Viet Nam
5. Hue University of Medicine and Pharmacy, Hue University, Viet Nam
6. Gia Lai Provincial Hospital, Viet Nam
7. Hanoi University of Pharmacy, Viet Nam
8. Viet Nam National Children’s Hospital, Viet Nam
9. Nuffield Department of Medicine, University of Oxford, UK

**Table S1. Revised Standards for Quality Improvement Reporting Excellence (SQUIRE 2.0)**

| **Text Section and Item Name** | **Section or Item Description** | **Response** |
| --- | --- | --- |
| **Title and Abstract** |  |  |
| 1. Title | Indicate that the manuscript concerns an initiative to improve healthcare (broadly defined to include the quality, safety, effectiveness, patient centeredness, timeliness, cost, efficiency, and equity of healthcare) | Page 1 |
| 2. Abstract | a. Provide adequate information to aid in searching and indexing b. Summarize all key information from various sections of the text using the abstract format of the intended publication or a structured summary such as: background, local problem, methods, interventions, results, conclusions | Page 2 |
| **Introduction** | **Why did you start?** |  |
| 3. Problem description | Nature and significance of the local problem | Page 4 |
| 4. Available knowledge | Summary of what is currently known about the problem, including relevant previous studies | Page 4-5 |
| 5. Rationale | Informal or formal frameworks, models, concepts, and/or theories used to explain the problem, any reasons or assumptions that were used to develop the intervention(s), and reasons why the intervention(s) was expected to work | Page 5 |
| 6. Specific aims | Purpose of the project and of this report | Page 6 |
| **Methods** |  |  |
| 7. Context | Contextual elements considered important at the outset of introducing the intervention(s) | Page 7-8 |
| 8. Interventions | a. Description of the intervention(s) in sufficient detail that others could reproduce it  b. Specifics of the team involved in the work | Page 8-12 |
| 9. Study of intervention(s) | a. Approach chosen for assessing the impact of the intervention(s)  b. Approach used to establish whether the observed outcomes were due to the intervention(s) | Page 8-12 |
| 10. Measures | a. Measures chosen for studying processes and outcomes of the intervention(s), including rationale for choosing them, their operational definitions, and their validity and reliability  b. Description of the approach to the ongoing assessment of contextual elements that contributed to the success, failure, efficiency, and cost  c. Methods employed for assessing completeness and accuracy of data | Page 8-12 |
| 11. Analysis | a. Qualitative and quantitative methods used to draw inferences from the data  b. Methods for understanding variation within the data, including the effects of time as a variable | Page 12-13 |
| 12. Ethical considerations | Ethical aspects of implementing and studying the intervention(s) and how they were addressed, including, but not limited to, formal ethics review and potential conflict(s) of interest | Page 13 |
| **Results** |  |  |
| 13. Results | a. Initial steps of the intervention(s) and their evolution over time (e.g., time-line diagram, flow chart, or table), including modifications made to the intervention during the project  b. Details of the process measures and outcome  c. Contextual elements that interacted with the intervention(s)  d. Observed associations between outcomes, interventions, and relevant contextual elements  e. Unintended consequences such as unexpected benefits, problems, failures, or costs associated with the intervention(s).  f. Details about missing data | Page 14-19 |
| **Discussion** |  |  |
| 14. Summary | a. Key findings, including relevance to the rationale and specific aims  b. Particular strengths of the project | Page 19-23 |
| 15. Interpretation | a. Nature of the association between the intervention(s) and the outcomes  b. Comparison of results with findings from other publications  c. Impact of the project on people and systems  d. Reasons for any differences between observed and anticipated outcomes, including the influence of context  e. Costs and strategic trade-offs, including opportunity costs | Page 19-23 |
| 16. Limitation | a. Limits to the generalizability of the work  b. Factors that might have limited internal validity such as confounding, bias, or imprecision in the design, methods, measurement, or analysis  c. Efforts made to minimize and adjust for limitations | Page 22-23 |
| 17. Conclusions | a. Usefulness of the work  b. Sustainability  c. Potential for spread to other contexts  d. Implications for practice and for further study in the field  e. Suggested next steps | Page 23-24 |
| **Other information** |  |  |
| 18. Funding | Sources of funding that supported this work. Role, if any, of the funding organization in the design, implementation, interpretation, and reporting | Page 25 |

**Checklist reference:** Ogrinc G, et al. SQUIRE 2.0 (Standards for Quality Improvement Reporting Excellence). BMJ Qual Saf. 2016;25:986–992.

Table S2. Timeline of antimicrobial stewardship (AMS) activities across participating hospitals, 2024–2025

| **Activity** | **NHTD** | **VSUH** | **GLPH** | **UMPH** |
| --- | --- | --- | --- | --- |
| Initiation meeting (online) | conducted under a previous project | conducted under a previous project | 18/12/2024 | 19/12/2024 |
| Follow-up meeting 1 (online) | 19/6/2025 | 25/4/2025 | 25/4/2025 | 10/3/2025 |
| Planning meeting (onsite, including SWOT analysis and overall implementation planning) | 17/6/2025 | 6/5/2025 | 29/4/2025 | 17/3/2025 |
| Baseline AMS assessment (G-ASET, ICARS, KAP form [electronic form in REDCap] sent to sites) | 11/8/2025 | 27/6/2025 | 30/5/2025 | 30/5/2025 |
| Follow-up meeting 2 (focused action planning) | 9/9/2025 (onsite) | 13/8/2025 (online) | 3/7/2025 (online) | 2/7/2025 (online) |
| AMS action plan drafted and approved by hospital director for 2025-2026 | 15/9/2025 | 16/9/2025 | 14/7/2025 | Draft completed: 30/9/2025  Official approval: 06/02/2026, delayed due to the appointment of a new board of directors |
| Follow-up AMS assessment (G-ASET, ICARS, KAP form [electronic form in REDCap] sent to sites) | 25/9/2025 | 25/9/2025 | 25/9/2025 | 25/9/2025 |
| Formal implementation of the approved AMS action plan | 9/2025 | 10/2025 | 7/2025 | 9/2025 |
| Ongoing implementation activities with continued technical support from OUCRU after completion of the Fleming funded project. | Development of AMS reports; development of antibiograms and digital platform (iSHARP) using routine AMR and HIS data | Audit and feedback | Audit and feedback; updated AMS and antibiotic use guidelines | Audit and feedback; updated AMS and antibiotic use guidelines; development of AMS reports; development of antibiograms using routine AMR data |

Table S3. Site-specific AMS-IPC quality improvement package and support provided, 2024–2026. Core sequence delivered: initiation, onsite planning, follow-up meetings, baseline assessment, and follow-up assessment;

| **Site** | **Baseline engagement** | **Planning and feedback meetings** | **Workshops delivered** | **Materials / SOPs provided** | **Action-plan status** | **Ongoing support after baseline** | **Site-specific deviations / delays** |
| --- | --- | --- | --- | --- | --- | --- | --- |
| **NHTD** | Existing site in OUCRU’s AMS research programme; prior AMS assessment experience and an established AMS programme with an IPC department/surveillance infrastructure | As planned, 3 core meetings conducted during this project | As planned, 4 workshops delivered for the whole network | Audit/feedback procedures; AWaRe materials; antibiogram template; AMS reporting template | AMS action plan approved for 2025–2026 | Development of AMS reports and antibiograms using routine AMR data; ongoing OUCRU support for digital platform for AMS-IPC (iSHARP) | Due to administrative delays, staff participation from NHTD was limited in the first two workshops. |
| **VSUH** | Existing site in OUCRU’s AMS research programme; prior AMS assessment experience and a formal AMS programme with variable implementation | As planned, 3 core meetings conducted during this project | As planned, 4 workshops delivered for the whole network | AMS SOPs and IEC materials; audit/feedback procedures; IV-to-PO guidance; authorized antibiotics; AWaRe materials; AMS reporting template; treatment guideline review/update | AMS action plan approved for 2025–2026 | Audit and feedback; continued OUCRU support as needed within the hospital network | No major deviation noted |
| **GLPH** | New site; no prior formal AMS or ICAR assessment; no formal AMS programme at baseline | As planned, 4 core meetings conducted during this project | As planned, 4 workshops delivered for the whole network | AMS SOPs and IEC materials; audit/feedback procedures; IV-to-PO guidance; authorized antibiotics; AWaRe materials; AMS reporting template; treatment guideline review/update | AMS action plan approved for 2025–2026 | Audit and feedback; updated AMS and antibiotic use guidelines | No major deviation noted |
| **UMPH** | New site; no prior formal AMS or ICAR assessment; partial stewardship activities embedded in practice at baseline | As planned, 4 core meetings conducted during this project | As planned, 4 workshops delivered for the whole network | AMS SOPs and IEC materials; audit/feedback procedures; IV-to-PO guidance; authorized antibiotics; AWaRe materials; antibiogram template; AMS reporting template; treatment guideline review/update | Draft completed for 2025–2026; official approval delayed to 06/02/2026 | Audit and feedback; updated AMS and antibiotic use guidelines; AMS reports; development of antibiograms; continued OUCRU support for microbiology and WHONET as needed | Approval delayed because of appointment of a new board of directors |

**Figure S1.** Visualisations of the KAP results were used to support discussion of targeted training priorities during AMS team meetings. At each timepoint, each hospital administered the KAP survey to approximately 50 staff members, including doctors, pharmacists, and microbiologists, with participation determined by staff availability at the time of assessment. The survey was based on the KAP form developed within the US CDC’s G-ASET toolkit [32,33]. The figures suggested small but consistent shifts towards more favourable responses at follow-up, particularly for attitude and practice items, although the magnitude and pattern of change varied across hospitals and several items remained unchanged. These observations were based on visual inspection rather than formal statistical inference and should therefore be interpreted cautiously. Nonetheless, the KAP surveys were useful in stimulating discussion and informing education and training activities to support programme implementation.

**Supplementary Method S1. Implementation of AMS in hospitals participating in the Fleming-funded project (2024–2025)**

At each participating hospital, a structured process was implemented to support the development and execution of antimicrobial stewardship (AMS) programmes (Table S2). This process began with baseline AMS assessments and initiation meetings with hospital leadership to identify existing capacities and gaps. Action-planning meetings, conducted both face-to-face and online, were then used to co-develop hospital-specific AMS plans, followed by regular follow-up meetings to review progress and address implementation challenges. Draft action plans were developed and internally reviewed before being finalized and formally approved through hospital decision letters. Once approved, the plans were disseminated to relevant departments to support implementation. Key AMS activities, including training, audit and feedback, and treatment guideline updates, were subsequently initiated. Implementation progress was continuously monitored and supported by the Oxford University Clinical Research Unit (OUCRU) Viet Nam through ongoing engagement aligned with priorities identified during the planning process.

***Initiation and planning meetings with AMS committees***

The research team conducted a series of structured engagement activities to assess and support AMS implementation at each site (Table S2). Initial meetings were held with hospital leadership and relevant stakeholders to review the status of AMS implementation and the functioning of AMS committees. From December 2024, two online meetings were conducted at each hospital: (1) a discussion with the coordination team and hospital leadership, and (2) a joint session with the coordination team and clinicians to introduce AMS activities and conduct a baseline AMS assessment. In addition, one in-person planning meeting was held at each hospital with either the hospital leadership and AMS committee or, where formal AMS structures were not yet established, with representatives from all departments.

AMS committees were encouraged to review ongoing AMS-related activities, including pharmacy reports, integrated plans, and infection prevention and control activities, and to conduct a self-assessed strengths, weaknesses, opportunities, and threats (SWOT) analysis for presentation and discussion during the meetings. Follow-up meetings were subsequently held to review progress and refine implementation plans. Hospitals were also advised to update AMS committee membership, clarify roles and responsibilities, and develop a one-year action plan (to be annually reviewed and updated). Expected outputs included hospital-specific AMS action plans for 2025–2026, focusing on key activities such as staff training, audit and feedback, standardized reporting, and updating treatment guidelines in line with revised national AMS guidance.

***AMS workshops***

Based on the outcomes of these discussions, OUCRU Viet Nam delivered four targeted workshops to address locally identified needs. Workshop content was informed by the priorities identified by AMS committees and teams, as well as findings from the knowledge, attitudes, and practices (KAP) survey. The workshops were delivered through online platforms, by recognized and experienced experts in the field in Vietnam from National Hospital for Tropical Diseases, Hanoi University of Pharmacy, and Vietnam Children Hospital. Participation rates were monitored, and engagement was assessed by the proportion of designated staff attending each workshop.

The poster of AMS online workshops*:

Workshop content:

| **Workshop 1**  16 May 2025 | **Workshop 2**  23 May 2025 | **Workshop 3**  06 June 2025 | **Workshop 4**  20 June 2025 |
| --- | --- | --- | --- |
| Antibiotic treatment in sepsis  AMS concepts and implementation guidelines, clinical pharmacy roles and activities  Roles of clinical microbiology in AMS  Standard sampling procedures and improving quality of clinical specimens  Understanding and interpreting culture results in clinical practices | Antibiotic treatment in community-acquired pneumonia  Multidrug-resistant infection caused by Gram-positive bacteria  Antibiotic dosing  Microbiologist -clinician communication  Antibiotic susceptibility testing and results reporting  Developing antibiogram reports | Antibiotic use in surgical prophylaxis  Antibiotic treatment of urinary tract infections  Multidrug-resistant infection caused by Gram-negative bacteria | Antibiotic treatment in common hospital-acquired infections caused by MRSA, VRE, CRE  Implementing antibiotic prescription based audit with feedback to prescribers |

Workshop attendance:

Overall, 363 healthcare workers from the four hospitals attended at least one training session, with at least 531 cumulative attendances recorded across all sessions. These figures are based on the number of Zoom users and may underestimate attendance, as some healthcare workers participated in groups using a shared Zoom connection. The number and professional composition of participants at each site are presented below.

| **Department** | **NHTD** | **VSUH** | **GLPH** | **UMPH** | **Total** |
| --- | --- | --- | --- | --- | --- |
| Internal medicine | 4 (8%) | 5 (13%) | 133 (60%) | 6 (11%) | 148 (41%) |
| Surgery | 5 (10%) | 16 (43%) | 64 (29%) | 8 (15%) | 93 (26%) |
| Infectious diseases | 5 (10%) | 1 (3%) | 5 (2%) | 0 (0%) | 11 (3%) |
| ICU | 14 (27%) | 1 (3%) | 4 (2%) | 9 (17%) | 28 (8%) |
| Pediatrics | 2 (4%) | 0 (0%) | 0 (0%) | 2 (4%) | 4 (1%) |
| Microbiology | 4 (8%) | 6 (16%) | 4 (2%) | 14 (26%) | 28 (8%) |
| Pharmacy | 4 (8%) | 5 (13%) | 2 (1%) | 9 (17%) | 20 (5%) |
| IPC | 1 (2%) | 0 (0%) | 0 (0%) | 0 (0%) | 1 (<1%) |
| Administration | 3 (6%) | 3 (8%) | 5 (2%) | 1 (2%) | 12 (3%) |
| Outpatient | 4 (8%) | 0 (0%) | 4 (2%) | 0 (0%) | 8 (2%) |
| Obstetrics & Gynecology | 1 (2%) | 0 (0%) | 0 (0%) | 5 (9%) | 6 (2%) |
| Director board | 4 (8%) | 0 (0%) | 0 (0%) | 0 (0%) | 4 (1%) |
| Total | 51 (100%) | 37 (100%) | 221 (100%) | 54 (100%) | 363 (100%) |

***Supportive documents and standard operating procedures***

Online resources outlining standard operating procedures (SOPs) for AMS activities were provided to all participating hospitals. These SOPs were updated in line with national policy, including Decision No. 5631/QD-BYT, and relevant international guidance, and were used as reference materials to support local implementation.

SOPs included:

- audit and feedback procedures linked to the antibiotic prescribing systems
- intravenous-to-oral switch (IV-to-PO) procedures
- lists of authorized antibiotics and approval processes
- AWaRe guidance
- AMS leaflets and posters
- an antibiogram template and development procedures
- an AMS reporting template for the four participating hospitals

AMS information, education, and communication (IEC) materials included:

- IV-to-PO posters
- materials on authorized antibiotic lists and approval processes
- AWaRe guideline posters and AWaRe classification lists
- educational leaflets and posters for doctors, patients, and caregivers

***Treatment guidelines***

Hospital AMS committees, in collaboration with OUCRU Viet Nam and the National Hospital for Tropical Diseases (NHTD), reviewed existing treatment guidelines and updated them where necessary to align with current best practices and local evidence. The revised guidelines were disseminated to participating hospitals. Hospitals were also strongly encouraged to proactively review and update their local treatment guidelines to support audit and feedback activities for antibiotic prescribing. All participating hospitals undertook such reviews and included the updating of key treatment guidelines in their annual action plans, including guidelines related to surgical antibiotic prophylaxis.

Table S4. Baseline summary of antibiotic use across four hospitals prior to the AMS-IPC quality improvement initiative

| **Hospital** | **Level/ type** | **Geographic location** | **Primary clinical focus** | **Baseline status of antibiotic use** |
| --- | --- | --- | --- | --- |
| NHTD | Tertiary referral hospital | North Viet Nam | Infectious diseases | At baseline (HIS data in 2022), NHTD showed a watch-dominant antibiotic-use pattern. Watch antibiotics accounted for 67% of total DOT, followed by Access (19%) and Reserve (9%). Ward-level use was highest in ICU (~1,800 DOT/1000 patient-days), emergency (~1,700), general surgery (~1,000), general infection (~800), and pediatrics (~700). The highest DOT/1,000 patient-days were seen with ticarcillin, ceftriaxone, imipenem/cilastatin, cefoperazone, ampicillin/sulbactam, cefuroxime, metronidazole IV, ertapenem, ciprofloxacin, and doxycycline, indicating substantial broad-spectrum inpatient prescribing, particularly in higher-acuity wards. |
| VSUH | Provincial general hospital | North Viet Nam | General acute care | At baseline (2022), VSUH showed a watch-dominant antibiotic-use pattern. On a point-prevalence survey day in December 2022, Watch antibiotics accounted for 61% of prescriptions, followed by Access (30%), with no Reserve antibiotics used and 0.4% unclassified. Ward-level use (from HIS data in 2022) was higher in pediatrics (~800 DOT/1000 patient-days), emergency (~600), and surgery (~650) than in other wards. Across most wards and months, Watch antibiotics predominated, while Access use was more prominent in surgical, paediatric, and obstetric/ gynaecology services. |
| GLPH | Provincial general hospital | Central Highlands | General acute care | At baseline (point-prevalence survey data in 2025), GLPH showed a broad-spectrum inpatient antibiotic-use pattern with a relatively balanced mix of Access and Watch antibiotics: Access (44% of total DOT), Watch (41%), and Reserve (13%). Ward-level use was highest in ICU (~1,700 DOT/1000 patient-days), obstetrics-gynaecology (~1,200), surgery (~1,100), pediatrics (950), and internal medicine (680). The most frequently used antibiotic groups over the first quarter of 2025 were beta-lactam/beta-lactamase inhibitor combinations, followed by third-generation cephalosporins, aminoglycosides, and fluoroquinolones. |
| UMPH | Academic general hospital | Central Viet Nam | General and teaching hospital | At baseline (pharmacy reports), UMPH showed substantial antibiotic use in inpatient services, with broad-spectrum beta-lactams appearing to contribute most to total antibiotic expenditure in the previous year. During the planning meeting, the hospital focused on selected higher-priority agents, including meropenem, vancomycin, imipenem/cilastatin, linezolid, fosfomycin, and colistin. The mean length of treatment for these agents ranged from 4 days for colistin to 10 days for vancomycin. |
